# Personality Traits Affect the Cost‐Effectiveness of Total Knee Arthroplasty

**DOI:** 10.1111/os.13017

**Published:** 2021-04-08

**Authors:** Chao Chen, Ying‐ying Shi, Xiao An, Long Gong, Ming‐sheng Tan, Zhi‐yuan Fang

**Affiliations:** ^1^ Department of Orthopaedics Beijing Hospital of Traditional Chinese Medicine, Capital Medical University Beijing China; ^2^ Department of Psychology Hainan Hospital of Chinese PLA General Hospital Sanya China; ^3^ Department of Orthopaedics Hainan Hospital of Chinese PLA General Hospital Sanya China; ^4^ Department of Orthopaedics, China‐Japan Friendship Hospital, Peking Union Medical College Chinese Academy of Medical College Beijing China; ^5^ Beijing University of Chinese Medicine Beijing China; ^6^ Dongfang Hospital Beijing University of Chinese Medicine Beijing China

**Keywords:** Cost‐effectiveness ratio, Eysenck Personality Questionnaire, Quality‐adjusted life years, Short‐Form 36, Total knee arthroplasty, Western Ontario and McMaster Universities questionnaire

## Abstract

**Objective:**

To assess the clinical benefit and compare the cost‐effectiveness of total knee arthroplasty (TKA) in patients with different personality traits.

**Methods:**

The present study was retrospectively conducted from January 2017 to May 2018. A total of 232 patients between 46 and 71 years old who underwent unilateral, primary TKA with the diagnosis of knee osteoarthritis were interviewed. Three types of data were required to compare the cost‐effectiveness differences among groups: personality traits, postoperative clinical outcomes about health‐related quality of life, and costs associated with TKA. Personality was assessed using the Eysenck Personality Questionnaire, functional outcome was assessed through the Western Ontario and McMaster Universities Osteoarthritis Index (WOMAC) questionnaire, and costs were evaluated. Besides, the marginal cost‐effectiveness ratio (MCER) as the primary outcome, which relates the direct costs to the associated patient benefit as assessed by the clinical endpoint ($/quality‐adjusted life years [QALY]), was compared among different personality traits. All information for this study was acquired by directly interviewing the patients and reviewing the medical computer records at our hospital.

**Results:**

Two hundred and eleven patients completed the final analysis with an average of 24.6 months follow‐up postoperatively. The choleric group, sanguine group, melancholic group, and phlegmatic group has 41, 70, 46, and 54 patients, respectively. A statistically significant difference in MECR, QALYs, and postoperative WOMAC existed among different personality traits (all *P* < 0.05). There was no significant difference in mean age (*P* = 0.588), body mass index (BMI) (*P* = 0.790), smoking (*P* = 0.934), heavy drinking (*P* = 0.994), chronic comorbidities (all *P* > 0.05), preoperative albumin <3.5 g/dL (*P* = 0.991), and American Society of Anaesthesiologists (ASA) score (*P* = 0.687) among personality traits. More women tend to be melancholic in comparison to other personality traits (*P* = 0.016). Melancholic patients attested inferiority of TKA compared with other personality traits, who would pay for the same QALYs at the highest costs (*P* < 0.05). By contrast, sanguine patients have a more cost‐effective TKA than other personality traits, as they pay the least money for the same QALYs (*P* < 0.05). Although phlegmatic and choleric patients seemingly have moderate gains from TKA, in general, the extroversion (measured by the extroversion subscale) and stability (measured by the neuroticism subscale) displayed more pleasurable QALYs in comparison with introversion and instability (*P* < 0.05). Sensitivity analysis showed that the results mentioned above appeared not to be sensitive when varying key parameters (prosthesis survival and life expectancy) in a one‐way sensitivity analysis. Sanguine and melancholic patients still have the lowest and highest MCER in comparison with choleric and phlegmatic traits (*P* < 0.05). The multivariate logistic regression showed that RA (adjusted *OR* = 1.3, 95% *CI* = 1.2–1.4, *P* < 0.01), ASA Class I–II (adjusted *OR* = 0.9, 95% *CI* = 0.8–1.0, *P* < 0.001), sanguine (adjusted *OR* = 0.8, 95% *CI* = 0.7–0.9, *P* < 0.001) and melancholic (adjusted *OR* = 1.2, 95% *CI* = 1.1–1.3, *P* < 0.001) were significantly associated with MCER.

**Conclusions:**

Before surgery, screening the melancholic patients would significantly reduce the economic burden, avoid unnecessary suffering, and shorten the recovery period.

## Graphical abstract

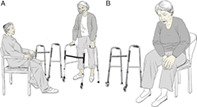

Melancholic personality is a severe problem for a good cost‐effectiveness ratio of TKA, producing high costs but less benefit on quality of life for the public health system. Before surgery, screening the melancholic patients would significantly reduce the economic burden, avoid unnecessary suffering, and shorten the recovery period.

## Introduction

Knee osteoarthritis (KOA), as degenerative joint disease, is accompanied by the progressive, complicated, and multifaceted characteristics of feelings of chronic pain^1^. Total knee arthroplasty (TKA) is regarded as a successful therapeutic option to treat chronically painful conditions created by the severe degenerate tibiofemoral joint^1^, ^2^. More than 500,000 TKAs are performed each year in the United States, and according to current trends, more than 3.5 mn knee arthroplasties will be done annually within the next 25 years^1^, ^2^. Patient‐reported outcome measures are increasingly used to assess TKA and are an essential marker for showing surgical success, not only for radiographic measurements and functional performance^1^. In general, we can evaluate, qualitatively and quantitatively, how much patients benefit from TKA by using Short‐Form 36 (SF‐36) and Western Ontario and McMaster Universities (WOMAC) questionnaire, which cover pain relief, functional recovery, and improvement in quality of life^3^. From these assessments we can see that factors such as biomechanics, implants, and surgical operation affect patient outcomes after TKA^2^. Besides, other factors, such as increased operative times, complications, and perioperative care, could prevent the clinical benefits and meanwhile increase hospital costs or patient charges^2^, ^3^. These factors are closely associated with the patient's satisfaction towards TKA.

In the last decade, several studies have demonstrated that personality traits may play a role in the interaction of TKA‐related disease processes such as function and health perception and affect the response and interpretation of psychometric and patient‐reported outcome measures^3^, ^4^, ^5^, ^6^, ^7^, ^8^, ^9^. To be specific, our previous study showed a negative relationship between outcome scores, especially from the SF‐36 Mental Component Summary (MCS), WOMAC pain scores, and neuroticism subscales scores. Sanguine patients displayed the best clinical outcomes among different personality traits, while melancholic patients showed the worst. Despite good clinical outcomes, including in pain relief and functional improvement for choleric patients, the satisfaction rate was unexpectedly the lowest^7^. Michele *et al*. indicated that personality traits and anxiety predict residual pain following total hip and knee arthroplasty; thus, preoperative evaluation of these factors could help identify patients at risk for residual pain^8^. Giurea *et al*. demonstrated dissatisfied and satisfied participants showed different levels of personality traits. Namely, personality traits could affect the patient's satisfaction and clinical outcome after TKA. Therefore, a patient's personality traits may be a useful predictive factor for postoperative satisfaction after TKA^5^. Matthias *et al*. showed that category of type D personality, defined as a general inclination to psychological distress, with the primary source of that distress being the components of type D personality, social inhibition, and negative effect, was related to the persisting pain, which was closely associated with persisting dysfunction after the TKA^9^. Based on these findings from previous studies, a patient's personality traits are crucial elements in the evaluation of TKA.

With the purpose of developing and maintaining a universal health insurance system, it is of great necessity to allocate finite medical resources effectively. When considering the allocation and utilization of these medical resources on TKA‐related diseases, it is expected that, going forward, health economics will be increasingly applied and highly valued. Besides, the principles and basis will be shared among the parties involved. In today's health care climate, the cost‐effectiveness analysis (CEA) has served as an integral part of a plausible intervention. It is of great significance for policymakers, insurers, researchers, and clinicians to assist health care resource allocation by conducting the CEA. Costs and utilities—i.e. effectiveness—are two crucial inputs into any CEA. In general, its estimation is derived from the data at the individual patient level, which is used for clinical studies. Accurate data is the expected value from wholly decreasing the uncertainty surrounding the cost‐effectiveness of a particular treatment. In the field of arthroplasty in orthopaedics, patients were generally treated in a standardized and routinized way; for instance, similar implants, surgical techniques, and perioperative agents were used. However, just exactly as the analysis mentioned above, patients, acting as the central role in the disease process, have diverse personalities and these personalities affect both the utilities and costs^4^, ^5^, ^6^.

Therefore, in the present study, we intend to: (i) assess the clinical benefit of TKA in patients with each personality trait; (ii) determine whether a particular personality trait would have additional expenditures and compare the cost‐effectiveness of TKA among different personality traits; and (iii) provide data for preventive measures to optimize the cost‐effectiveness of TKA and lower costs in the population at risk for worse cost‐effectiveness.

To our knowledge, this is the first study to analyze the CEA outcomes after TKA from the perspective of patient personality traits. We hypothesized that there are differences in the cost‐effectiveness of TKA among personality traits; the Eysenck Personality Questionnaire (EPQ) showed that sanguine patients and melancholic patients showed superiority and inferiority to the effectiveness of TKA, respectively, in comparison to the other two personality traits.

## Materials and Methods

### Study Design

We intended to compare the difference in cost‐effectiveness among groups based on personality traits by adopting a real‐world data approach instead of implementing a Markov decision analysis based on a meta‐analysis.

The present study was retrospectively conducted from January 2017 to May 2018. The study was approved by the institutional review board of the authors’ affiliated institution and following the Declaration of Helsinki. Three types of data were required to compare the cost‐effectiveness differences among groups: personality traits, postoperative clinical outcomes about health‐related quality of life, and costs associated with TKA. All information for this study was acquired by directly interviewing the patients and reviewing the medical computer records at our hospital.

### Patient Characteristics

A total of 232 patients between 46 and 71 years old was interviewed. Patients were informed about the purposes of this retrospective health economic study and gave their written consent.

Inclusion criteria for the case group included: (i) the diagnosis of knee osteoarthritis following the International Classification of Diseases 9th Revision (ICD‐9); (ii) undergoing primary and unilateral TKA under epidural anesthesia; (iii) using the Gemini MK‐II (Link, Ltd., Germany); (iv) had completed data including clinical evaluation and examination used for comparison; (v) a retrospective study.

Exclusion criteria included: (i) extra medical costs (but not directly resulting from the psychological factors we studied) during the study period; (ii) malignant diseases, *severe* extra‐articular deformity, traumatic arthritis, chronic cardiovascular and cerebrovascular diseases, diabetes; (iii) postoperative infection; and (iv) other serious diseases and complications.

### Operative Procedure

All TKAs were performed by one surgical team in the respective institution using epidural anesthesia. Cruciate‐retaining and cementless prosthesis (CR) (Gemini MK‐II, Link, Germany) with patellar resurfacing were used. CR prostheses were implanted in the knees with mild to moderate deformities (flexion contracture and varus deformity of less than 15°) and an intact posterior cruciate ligament (PCL). The drainage tube was promptly extracted depending on the drainage volume within the first 24 h postoperatively. The pneumatic tourniquet was used with pressure at 55–75 kPa for about 40 min.

All patients were routinely given prophylactic cefuroxime 30 min preoperatively and 24 h postoperatively (1.5 g, iv, tid). Preventive anticoagulant therapy (10 mg rivaroxaban every day or 2850 international units [IU] low‐molecular‐weight heparin [LMWH] [body weight < 90 kg] or 5700 IU [body weight > 90 kg]) began within 12 h postoperatively and continued for 28 days at least.

Patients were encouraged to start weight‐bearing as soon as tolerable with ambulatory aids (usually within the first 24 h). They then were allowed to discontinue the assistance of aids as they could ambulate without a limp (generally within 6–12 weeks). The physical therapist performed a functional exercise depending on the functional performance after TKA. The postoperative X‐ray alignment on standard views of all enrolled cases has a proper lower limb alignment in weight‐bearing and non‐weight‐bearing positions with a good quality of component placement.

### Clinical Data

#### Personality Trait

**Fig. 1:**
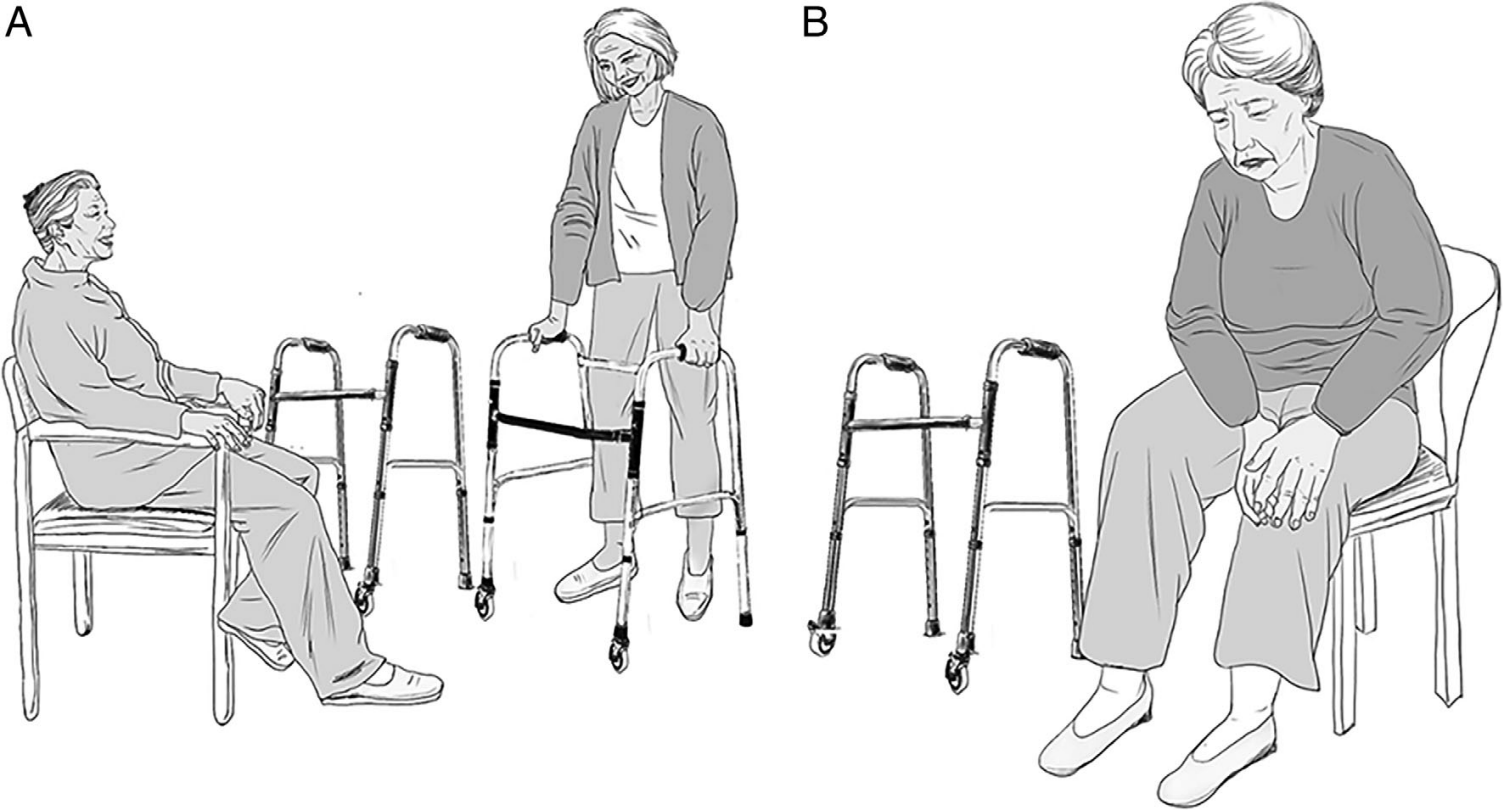
A drawing of the functional exercises of sanguine patients and melancholic patients undergoing total knee arthroplasty. (A) Sanguine patients tend to be sociable and optimistic, share their feelings with others, and obtain more information about rehabilitation. (B) Melancholic patients are inclined to be depressed and catastrophizing. Meanwhile, they are less talkative and therefore suffer more pain during the recovery from TKA.

In this study, each patient's personality trait was determined 1 week before with the help of the EPQ^7^. We used a Chinese translation of the original English version of the EPQ developed by Eysenck and colleagues. The EPQ has 48 questions with dichotomized answers for four personality subscales: extroversion, neuroticism, psychoticism, and lying. Higher scores indicate a greater tendency to possess one of these personality traits from 0 to 12. Extroversion represents sociability, liveliness, and surgency; neuroticism represents emotional instability and anxiousness; psychoticism represents tough‐mindedness, aggressiveness, coldness, and egocentricity; lying represents unsophisticated dissimulation^10^. According to the levels of extroverted/introverted (measured by the extroversion subscale) and stable/unstable (measured by the neuroticism subscale)^10^, patients were divided into four personality types: choleric, sanguine, melancholic, and phlegmatic (Fig. 1). This process was determined by a senior psychiatrist according to the previous criteria, which has demonstrated its consistency^10^. We evaluated test–retest reliability of this instrument from 30 participants who repeated the answers of the Chinese translation of the original English version of the EPQ twice at 1‐week intervals.

#### TKA‐Related Medical Cost

The total cost of each TKA in this study was defined as the sum of the costs of the following items: surgical procedures (including the occupation of the operating theater by the surgical team, the anesthetist, prosthetic material, and drugs), days of stay in the postoperative recovery unit, and, in the orthopaedic surgery ward, outpatient sessions (including checkups/surgical incision care), clinical tests ordered to confirm the diagnosis, drugs (mainly including analgesics and anticoagulants), complementary rehabilitation sessions during the first 3 months (paid by the hour), complications (mainly including deep venous thrombosis, periprosthetic infection, periprosthetic fracture, sepsis, etc.), and outpatient care. All costs were reported in 2018 US dollars ($).

#### Clinical Benefit

The primary outcome of this study was the marginal cost‐effectiveness ratio (MCER), which relates the direct costs to the associated patient benefit as assessed by the clinical endpoint. The clinical benefit in this study was measured in the form of quality‐adjusted life years (QALYs) before and 12 months after surgery, as assessed using the Western Ontario and McMaster Universities Osteoarthritis Index (WOMAC) questionnaire, describing the percentage of a patient's theoretical rest‐of‐life expectancy. QALYs quantitatively describe the clinical benefit obtained by the patients. The life expectancy distribution of the included population was assumed with a mean expectancy of 79 years for women and 75 years for men (both underlying standard deviations of 10 years) according to the demographic and health surveys^11^. In this study, we hypothesized that: (i) the rest‐of‐life expectancy of each patient was simulated; (ii) the functional outcome was assumed to persist unchanged over the rest of the patient's life; and (iii) loss of QALYs was considered the same when a revision became necessary. To account for the time‐dependent loss in the primary clinical benefit as assessed shortly after surgery, the overall benefit estimate was discounted at an annual discounting rate of 3%^12^. Also, we assumed that 5% of the patients per 10 years would accept the revision, as there was a 10‐year revision rate of around 5%–10% after TKA^13^, ^14^.

### Sample Size

The sample size was calculated to detect a mean difference in MCER of 500 $/QALYS with a standard deviation (SD) of 250. The error was set at 0.05 and the power level was at 90% with additional compensation for a possible dropout rate of 20%. The required sample size was 35 patients in each group at least.

### Sensitivity Analysis

The robustness of the cost‐effectiveness ratio (CER) crucially depends on the validity and precision of the following data: the rest‐of‐life expectancy of patients and the survival of the prosthesis. Therefore, we performed deterministic one‐way sensitivity analysis (systematically varying these two parameters over plausible ranges, respectively) to illustrate the relative impact of each individual assumption on the CER of TKA in each personality. The parameter ranges used in the one‐way sensitivity analyses were derived from the published literature. We used a variety of ±20% of the base case value when data were absent. Specifically, prosthesis survival and rest‐of‐life expectancy of patients varied deterministically by over ±3% (2%–8%) and ±5 years.

### Statistical Analysis

We used the median and semi‐interquartile range, or mean and standard deviation, to demonstrate the continuous variables. Ordinal variables were described as proportions. The chi‐square test, Student's *t*‐test, and Tukey's *post hoc* were carried out to compare variables in different personalities. The univariate logistic regression was performed to determine the association between MCER and personality traits. The covariates were analyzed in the multivariate logistic regression if the crude odd ratio was statistically significant. Different personality traits were input as categorical independent variables (choleric as reference). The statistical significance and power analysis were *P*‐value ≤0.05 and 0.8, respectively. The internal consistency reliability was measured using Cronbach's alpha: previous studies suggest that Cronbach's alpha >0.5 is considered acceptable reliability^15^, ^16^. The intraclass correlation coefficient (ICC) was used to examine one‐week test–retest reliability of EPQ questionnaire. Acceptable test–retest reliability was greater than 0.75^17^. SPSS version 22 (SPSS; Chicago, IL, USA) was used to perform all analyses.

## Results

### General Results

Of 232 patients who responded to both the EPQ and WOMAC questionnaire, 21 were excluded after review: four patients who had had severe diseases during the observation period and seven patients with severe complications after TKA (two patients were PJI; one patient was sepsis; four patients were DVT); 211 patients remained in the final analysis. The choleric group, sanguine group, melancholic group, and phlegmatic groups comprised of 41, 70, 46, and 54 patients, respectively. The baseline characteristics of the patient population are detailed in Table 1. The patients were followed up for an average of 24.6 months postoperatively.

**TABLE 1:** Baseline characteristics of the patient population

Variables	Choleric	Sanguine	Melancholic	Phlegmatic	*F*/Chi square value	*P*‐value
Number of patients	41	70	46	54		—
Mean age (years)	62.3(5.3)	61.8(6.1)	60.4(5.7)	63.2(5.5)	2.058	0.107
Sex (Male)(*n*, %)	26(63.4%)	37(52.9%)	14(30.4%)	28(51.9%)	10.293	0.016
BMI (Kg/m^2^) (Mean, SD)	27.1(3.1)	26.8(2.8)	27.2(3.2)	27.0(3.0)	0.187	0.905
Smoking history(cases)	10(24.4%)	19(27.1%)	12(26.1%)	13(24.1%)	0.191	0.979
Heavy drinking(cases)	4(9.8%)	8(11.4%)	5(10.9%)	6(11.1%)	0.078	0.994
Hypertension(cases)	11(26.8%)	20(28.6%)	12(26.1%)	14(25.9%)	0.425	0.935
Cardiac diseases(cases)	5(12.2%)	10(14.3%)	6(13.0%)	7(13.0%)	0.110	0991
COPD(cases)	4(9.8%)	6(8.6%)	4(8.7%)	5(9.3%)	0.054	0.997
RA(cases)	5(12.2%)	10(14.3%)	6(13.0%)	7(13.0%)	0.110	0.991
Diabetes mellitus(cases)	8(19.5%)	16(22.9%)	10(21.7%)	10(18.5%)	0.415	0.937
Preoperative albumin <3.5 g/dL(cases)	3(7.3%)	5(7.1%)	4(8.7%)	4(7.4%)	0.107	0.991
ASA I–II(cases)	39(95.1%)	68(97.1%)	43(93.5%)	50(92.6%)	1.481	0.687

ASA, American Society of Anaesthesiologists; BMI, Body Mass Index; COPD, chronic obstructive pulmonary disease; RA, Rheumatology Arthritis; SD, standard deviation.

Values in parentheses are standard deviation. One‐way ANOVA test or chi‐square used for the statistical analysis; *P*‐value significant at 0.05.

There were no significant differences in mean age (62.3 ± 5.3 *vs* 61.8 ± 6.1 *vs* 60.4 ± 5.7 *vs* 63.2 ± 5.5, *P* = 0.588), BMI (27.1 ± 3.1 *vs* 26.8 ± 2.8 *vs* 27.2 ± 3.2 *vs* 27.0 ± 3.0, *P* = 0.790), percentage of smoking (24.4% *vs* 27.1% *vs* 26.1% *vs* 24.1%, *P* = 0.934), heavy drinking (9.8% *vs* 11.4% *vs* 10.9% *vs* 11.1%, *P* = 0.994), hypertension (26.8% *vs* 28.6% *vs* 26.1% *vs* 25.9%, *P* = 0.987), cardiac diseases (12.2% *vs* 14.3% *vs* 13.0% *vs* 13.0%, *P* = 0991), COPD (9.8% *vs* 8.6% *vs* 8.7% *vs* 9.3%, *P* = 0997), RA (12.2% *vs* 14.3% *vs* 13.0% *vs* 13.0%, *P* = 0.991), diabetes mellitus (19.5% *vs* 22.9% *vs* 21.7% *vs* 18.5%, *P* = 0.937), preoperative albumin <3.5 g/dL (7.3% *vs* 7.1% *vs* 8.7% *vs* 7.4%, *P* = 0.991), and ASA ≤II (95.1% *vs* 97.1% *vs* 93.5% *vs* 92.6%, *P* = 0.687) in choleric group, sanguine group, melancholic group, and phlegmatic group. More women tended to be melancholic in comparison to choleric, sanguine, or phlegmatic (30.4% *vs* 63.4% *vs* 52.9% *vs* 51.9%, *P* = 0.016).

### Reliability

Cronbach's alpha coefficient of Chinese translation of the original English version of the EPQ was 0.81, and each item ranged from 0.80 to 0.83. The intraclass correlation coefficient (*ICC*) ranged from 0.81 to 0.85.

### Base Case Analyses

As for QALYs (5.8 ± 1.1 *vs* 6.9 ± 1.3 *vs* 5.0 ± 1.2 *vs* 5.6 ± 1.2), postoperative WOMAC (42.1 ± 8.5 *vs* 37.9 ± 9.2 *vs* 51.7 ± 8.2 *vs* 43.2 ± 8.8), change of total WOMAC scores (71.1 ± 9.3 *vs* 77.6 ± 11.3 *vs* 65.8 ± 8.3 *vs* 71.2 ± 9.2) and MECR (2728.2 ± 478.4 *vs* 2264.0 ± 421.6 *vs* 3504.7 ± 576.2 *vs* 2821.5 ± 500.5) in choleric group, sanguine group, melancholic group and phlegmatic group, there were statistically significant differences among any two different personality traits (all *P*<0.05), except between choleric and phlegmatic group (*P* > 0.05, Table 2).

**TABLE 2:** Base case analyses for different personality traits

Variables	Pre‐operation WOMAC	Post‐operation WOMAC	Change of total WOMAC scores	QALYS	MCER($/QALYS)
Choleric	113.2 (11.2)	42.1 (8.5)	71.1 (9.3)	5.8 (1.1)	2728.2 (478.4)
Sanguine	115.5 (10.3)	37.9 (9.2)	77.6 (11.3)	6.9 (1.3)	2264.0 (421.6)
Melancholic	117.5 (8.5)	51.7 (8.2)	65.8 (8.3)	5.0 (1.2)	3504.7 (576.2)
Phlegmatic	114.4 (9.5)	43.2 (8.8)	71.2 (9.2)	5.6 (1.2)	2821.5 (500.5)
*P*‐value	0.211	<0.001	<0.001	<0.001	<0.001

Values in parentheses are standard deviation. One‐way ANOVA test used for the statistical analysis; *P* value significant at 0.05. *means there was statistically significant between‐group difference.

According to this result, sanguine group had the highest QALYs and change of total WOMAC scores but had the lowest postoperative WOMAC and MCER in comparison with other traits (all *P* < 0.05).

Melancholic group had the lowest QALYs and change of total WOMAC scores but had the highest postoperative WOMAC and MCER in comparison with other traits (all *P* < 0.05).

### Sensitivity Analysis

As for MCER in base analysis in choleric group, sanguine group, melancholic group, and phlegmatic group (2728.2 ± 478.4 *vs* 2264.0 ± 421.6 *vs* 3504.7 ± 576.2 *vs* 2821.5 ± 500.5), there were statistically significant differences among any two different personality traits (all *P* < 0.05), except between choleric and phlegmatic group (*P* > 0.05, Table 3).

**TABLE 3:** One‐way sensitivity analysis in different personality traits

Variables	Base case	Rest life expectancy of patients+5 years	Rest life expectancy of patients‐5 years	Prosthesis survival+ 3%	Prosthesis survival‐ 3%
Choleric	2728.2 (478.4)	2600.5(480.2)	2850.4 (485.3)	2530.2 (464.2)	2908.3 (481.5)
Sanguine	2264.0 (421.6)	2136.3(430.5)	2390.2(425.5)	2022.3 (412.4)	2445.6 (432.4)
Melancholic	3504.7 (576.2)	3362.4 (570.2)	3650.7(573.3)	3302.3 (552.3)	3680.6 (562.3)
Phlegmatic	2821.5 (500.5)	2703.5 (511.2)	2940.5 (502.4)	2611.2 (512.3)	3002.6 (504.2)
*P*‐value	<0.001	<0.001	<0.001	<0.001	<0.001

Data representing cost‐effectiveness (MCER) results of the sensitivity analyses. Values in parentheses are 95% confidence interval. One‐way ANOVA test used for the statistical analysis; *P* value significant at 0.05. *means there was statistically significant between‐group difference.

Base analysis showed that sanguine group still had the highest QALYs and change of total WOMAC scores but had the lowest postoperative WOMAC and MCER in comparison with other traits (all *P* < 0.05). Melancholic group still had the lowest QALYs and change of total WOMAC scores, but had the highest postoperative WOMAC and MCER in comparison with other traits (all *P* < 0.05). The results appeared not to be sensitive when varying prosthesis survival and life expectancy in a one‐way sensitivity analysis, as illustrated in Table 3.

### Cost Details

As for all the costs, including hospital stay (440.2 ± 70.5 *vs* 401.2 ± 69.2 *vs* 696.6 ± 80.2 *vs* 431.2 ± 92.1), medical treatment (2502.3 ± 393.2 *vs* 2322.2 ± 360.5 *vs* 3210.2 ± 400.2 *vs* 2531.4 ± 380.2), rehabilitation (2320.5 ± 379.2 *vs* 2132.5 ± 362.1 *vs* 3600.4 ± 413.5 *vs* 2321.3 ± 354.5), outpatient care (check‐up: 712.5 ± 220.2 *vs* 687.5 ± 190.2 *vs* 1212.5 ± 212.2 *vs* 777.5 ± 198.2; traveling expenses: 1074.3 ± 321.2 *vs* 805.2 ± 266.4 *vs* 1713.2 ± 421.5 *vs* 952.7 ± 282.4), and total costs (15,823.4 ± 521.5 *vs* 15,081.6 ± 501.0 *vs* 17,523.6 ± 682.3 *vs* 15,800.6 ± 523.2), except surgical procedures (2728.2 ± 478.4 *vs* 2264.0 ± 421.6 *vs* 3504.7 ± 576.2 *vs* 2821.5 ± 500.5) in choleric group, sanguine group, melancholic group, and phlegmatic group, there were statistically significant differences among any two different personality traits (all *P*<0.05), except between choleric and phlegmatic group (*P*>0.05, Table 3).

As for the costs of surgical procedures in choleric group, sanguine group, melancholic group, and phlegmatic group (2728.2 ± 478.4 *vs* 2264.0 ± 421.6 *vs* 3504.7 ± 576.2 *vs* 2821.5 ± 500.5), there was no statistically significant difference (*P* = 0.368, Table 4).

**TABLE 4:** Cost details of each TKA in different personality traits

Variables	Surgical procedures	Hospital stay	Clinical tests/diagnostic tests	Medical treatment	Rehabilitation	Outpatient care		
						Check‐up	Traveling expenses	Total costs
Choleric	8432.3(212.3)	440.2(70.5)	350.3(110.2)	2502.3(393.2)	2320.5(379.2)	712.5(220.2)	1074.3(321.2)	15823.4 (521.5)
Sanguine	8423.5(200.5)	401.2(69.2)	309.5(97.4)	2322.2(360.5)	2132.5(362.1)	687.5(190.2)	805.2(266.4)	15081.6 (501.0)
Melancholic	8490.5(210.2)	696.6(80.2)	600.2(100.2)	3210.2(400.2)	3600.4(413.5)	1212.5 (212.2)	1713.2(421.5)	17523.6 (682.3)
Phlegmatic	8432.3(223.4)	431.2(92.1)	363.2(79.2)	2531.4(380.2)	2321.3(354.5)	777.5 (198.2)	952.7(282.4)	15800.6 (523.2)
*P*‐value	0.368	<0.001	<0.001	<0.001	<0.001	<0.001	<0.001	<0.001

Data representing costs. Values in parentheses are 95% confidence interval. One‐way ANOVA test used for the statistical analysis; *P* value significant at 0.05. *means there was statistically significant between‐group difference.

Sanguine group had the lowest in all costs except surgical procedures, while melancholic group had the highest costs (all *P* < 0.05).

### The Univariate Logistic Regression for the Association Between MCER and Clinical Variables

Univariate logistic regression analysis demonstrated that smoking history (adjusted *OR* = 1.1, 95% *CI* = 1.0–1.2, *P* = 0.01), cardiac diseases (adjusted *OR* = 1.3, 95% *CI* = 1.2–1.4, *P* < 0.01), COPD (adjusted *OR* = 1.1, 95% *CI* = 1.1–1.2, *P* < 0.01), RA (adjusted *OR* = 1.4, 95% *CI* = 1.3–1.5, *P* < 0.01), and melancholic personality (adjusted *OR* = 1.3, 95% *CI* = 1.2–1.4, *P* < 0.001) were significantly associated with a higher MCER, after adjusting for some confounders such as age, gender, and BMI. By contrast, ASA Class I–II (adjusted *OR* = 0.8, 95% *CI* = 0.7–0.9, *P* < 0.001) and sanguine (adjusted *OR* = 0.8, 95% *CI* = 0.7–0.9, *P* < 0.001) were significantly associated with a lower MCER when using ASA Class ≥ III and choleric trait as the references (Table 5).

**TABLE 5:** The univariate logistic regression for the associations between MCER and clinical variables

Variables	*OR*	*P*	Adjusted *OR* ^#^	*P*
Smoking history	1.2 (1.1–1.3)	<0.01	1.1 (1.0–1.2)	0.01
Heavy drinking	1.1 (1.0–1.2)	0.763	1.0 (1.0–1.1)	0.253
Hypertension	1.2 (1.1–1.3)	0.234	1.1 (1.0–1.2)	0.143
Cardiac diseases	1.4 (1.3–1.5)	<0.01	1.3 (1.2–1.4)	<0.01
COPD	1.2 (1.1–1.3)	<0.01	1.1 (1.1–1.2)	<0.01
RA	1.3 (1.2–1.4)	<0.01	1.4 (1.3–1.5)	<0.01
Diabetes mellitus	1.2 (1.1–1.3)	0.463	1.1 (1.0–1.2)	0.245
Preoperative albumin <3.5 g/dL	1.2 (1.1–1.3)	0.873	1.1 (1.0–1.2)	0.854
ASA Class I–II	0.8 (0.7–0.9)	<0.001	0.9 (0.8–1.0)	<0.001
Personality traits				
Choleric (ref.)				
Sanguine	0.7 (0.6–0.8)	<0.001	0.8 (0.7–0.9)	<0.001
Melancholic	1.4 (1.3–1.5)	<0.001	1.3 (1.2–1.4)	<0.001
Phlegmatic	1.1 (1.0–1.2)	0.232	1.0 (1.0–1.1)	0.463

*indicates statistically significant. ^#^ fully adjusted by confounding factors. Values in parentheses are 95% Confidence Interval. ASA, American Society of Anaesthesiologists; COPD, chronic obstructive pulmonary disease; OR, Odds Ratios; RA, Rheumatology Arthritis. *P*‐value significant at 0.05. *means there was statistically significant between‐group difference.

### Multivariate Logistic Regression for the Association Between MCER and Personality Traits

The factors that were significantly associated with MCER were further analyzed in the multivariate regression analysis. As demonstrated in Table 6, RA (adjusted *OR* = 1.3, 95% *CI* = 1.2–1.4, *P* < 0.01), ASA Class I–II (adjusted *OR* = 0.9, 95% *CI* = 0.8–1.0, *P* < 0.001), sanguine personality (adjusted *OR* = 0.8, 95% *CI* = 0.7–0.9, *P* < 0.001), and melancholic personality (adjusted *OR* = 1.2, 95% ***C**I* = 1.1–1.3, *P* < 0.001) remained significantly associated with MCER, after adjusting for some confounders such as age, gender, and BMI.

**TABLE 6:** The multivariate logistic regression for the associations between MCER and clinical variables

Variables	*OR*	*P*	Adjusted *OR* ^#^	*P*
Smoking history	1.3 (1.2–1.4)	0.865	1.2 (1.1–1.3)	0.834
Cardiac diseases	1.4 (1.3–1.5)	0.645	1.3 (1.2–1.4)	0.263
COPD	1.3 (1.2–1.4)	0.546	1.2 (1.1–1.3)	0.534
RA	1.4 (1.3–1.5)	<0.01	1.3 (1.2–1.4)	<0.01
ASA Class I–II	0.8 (0.7–0.9)	<0.001	0.9 (0.8–1.0)	<0.001
Personality traits				
Choleric (ref.)				
Sanguine	0.7 (0.6–0.8)	<0.001	0.8 (0.7–0.9)	<0.001
Melancholic	1.3 (1.2–1.4)	<0.001	1.2 (1.1–1.3)	<0.001

*indicates statistically significant. ^#^ fully adjusted by confounding factors. Values in parentheses are 95% Confidence Interval. ASA, American Society of Anaesthesiologists; COPD, chronic obstructive pulmonary disease; OR, Odds Ratios; RA, Rheumatology Arthritis. *P*‐value significant at 0.05. *means there was statistically significant between‐group difference.

## Discussion

### QALYs and MCER

Considering evaluating the effectiveness of the TKA procedure from the perspective of both clinical benefit and economic aspects benefits medical policymakers, doctors, and patients, this study intended to quantify the clinical benefit in terms of quality of life improvement and the individual cost/benefit relation of TKA. The primary purpose of the cost‐effectiveness evaluation was to derive a comparative cost‐effectiveness characteristic of TKA for different personalities and thereby provide rationales for recent discussions on resource allocation to arthroplasty and even other orthopaedic surgery. Our results showed the four personality types differed in postoperative WOMAC scores, but did not differ in the preoperative score. Accordingly, they proved clinically relevant and statistically significant differences in the WOMAC based benefit estimate. After transformation into the number of gained quality‐adjusted life years, the respective QALYs remain statistically significant between groups. The highest and lowest MCER for TKA were 3504.7 (576.2) $/QALY in melancholic patients and 2264.0 (421.6) $/QALY in sanguine patients, respectively. From the perspective of the health care insurer, the same medical inputs brought sanguine patients most QALYs but melancholic patients least ones than other personality traits. Although phlegmatic and choleric patients seemingly have moderate gains from TKA, in general, the extroversion (measured by the extroversion subscale) and stability (measured by the neuroticism subscale) displayed more pleasure QALYs in comparison with introversion and instability.

### Different Personality Traits and TKA

Extroversion represents sociability, liveliness, and surgency; neuroticism represents emotional instability and anxiousness^10^. Previous studies have demonstrated that patients who are talkative, easygoing, cooperated, and sociable tend to actively perform functional exercise with good and continued adherence to treatment and training tasks from doctors and rehabilitation practitioners^14^, ^18^, ^19^, ^20^, ^21^, ^22^. In contrast, those who were passive, controlled, depressive, and even pessimistic are inclined to be depressed and catastrophizing and thus need longer times to achieve postoperative functional targets such as 90° flexion^5^, ^18^, ^23^. Briefly, this could help explain why extroverted patients (choleric and sanguine types), especially those who are also stable (sanguine types), would have a better self‐reported outcome at lower costs when compared with those who are introverted (melancholic and phlegmatic type). In general, our results revealed the psychological dimension of extroversion determined the CER of TKA for one patient.

To briefly illustrate the estimation and interpretation of the MCER endpoint and its association with personality traits, a case included in this study is introduced: a 54‐year‐old female patient whose personality was determined as melancholic type complained about ongoing moderate pain persisting for 1 year after TKA. The possible causes, such as infection, malposition, and prosthetic loosening, had been excluded separately. Her over‐sensitiveness and doubt over treatments meant that she was hospitalized for nearly 2 weeks after the TKA, and had repeated consultations, checkups, and laboratory examinations 1 year postoperatively. Considering her complaint, her symptoms were mitigated by psychological interventions once a week and antidepressants provided by an experienced psychiatrist. The course of diagnosis and treatment required extra payment funded by her health care provider and herself. Besides, this patient experienced an unsatisfactory improvement in the quality of life. Therefore, it is of great benefit for both health care providers to avoid high cost‐effectiveness ratios. This is especially true for melancholic patients they tend to become anxious more easily before surgery, and hence it is necessary to take preventive measures to comfort or reassure their concerns about TKA surgery, such as elaborate preoperative conversations, cognitive therapy, and anti‐depressive medicine (if necessary) before TKA.

### Costs of the TKA in Different Personality Traits

The costs concerned in this study involved two main domains: TKA‐related costs in hospitals and costs from rehabilitation and outpatient care after discharge. In general, the cost‐effectiveness analysis regarding TKA is performed using the Markov decision model. It is an iterative process that the patients are assumed to stay in one cycle for a certain time and then make a transition to another cycle^20^, ^21^. Of note, exactly as the case mentioned above, the time and process of the transition could be influenced by a patient's personality trait. Previous findings further demonstrated that personality traits directly reflecting socialization, somatic distress, health worries, and performance orientation had been shown to affect the recovery after TKA^5^, ^6^, ^21^, ^22^.

Although several studies reach an agreement that personality traits may influence patient satisfaction and clinical outcome after TKA and it could serve as a useful predictive factor for postoperative satisfaction and recovery after TKA^5^, ^6^, ^22^, different measures for personality traits were used in a respective study. Giurea *et al*. investigated the personality parameters using the Freiburg Personality Inventory‐Revised (FPI‐R)^5^. This multi‐dimensional validated personality form consists of two dimensions of personality (social orientation and performance orientation), and 10 traits (inhibition, excitability, aggressiveness, strain, somatic distress, health worries, openness, extroversion, and emotional stability), and requires answering 138 questions by self‐evaluation. Mercurio *et al*. assessed personality traits using the revised Temperament and Character Inventory (TCI‐R)^8^. The questionnaire comprises 240 items covering four temperamental dimensions (novelty seeking, harm avoidance, reward dependence, and persistence) and three‐character dimensions (self‐directedness, cooperativeness, and self‐transcendence). Vogel *et al*. applied the Inventory of Personality Organization‐16 (IPO‐16), comprising the subscales identity diffusion (IPO‐ID), primitive defenses (IPO‐PD), reality testing (IPO‐RT), and the total score (IPO‐total), which is a 136‐item self‐report measure, and all items have a five‐point Likert‐type format, to determine the personality of patients undergoing TKA^6^. By comparison, we used the Eysenck personality questionnaire, which merely comprised 48 questions with dichotomized answers. This test has good internal consistency, test–retest reliability, and concurrent validity^22^. It can be performed easily with less time and is more friendly for both practitioners and patients in comparison with another three measures.

### Implications

A strength of the present study is the real‐world approach we used to determine the association between personality traits and CER. It was firstly introduced into the evaluation of TKA. This cost‐effectiveness study would be useful in assisting policymakers in identifying which patients can benefit less, formulate better medical policies, and alleviate vulnerable groups’ medical problems more effectively. It is also exceedingly meaningful for clinicians to grasp this information. Our study revealed that melancholic personality is a severe problem for a good CER of TKA, producing high costs but less benefit on quality of life for the public health system. Before surgery, screening the melancholic patients would significantly reduce the economic burden, avoid unnecessary suffering, and shorten the recovery period. Improved CER measures are needed to optimize the economic impact on health systems. These findings warrant replication and do upgrade our understanding of the psychosomatic implications of orthopaedic surgery. This indicated that we should highlight individualized treatment and change to a bio‐psycho‐social medical model.

### Limitations

Certain limitations should be acknowledged. A significant weakness is the retrospective cohort design in only one hospital. The sample size was limited. Second, since the out‐of‐pocket costs of subsequent treatment after discharge and other non‐treatment were not recorded in our database, we merely reviewed each patient's medical records. The results could be less persuasive. Third, more women tend to be melancholic, which is consistent with previous studies about the association between gender and personality traits^23^, ^24^. The gender could affect daily routine, lifestyle, and preference for treatment plans, which are closely associated with costs. Therefore, it could be possible that our observation of the differences in terms of outcomes were caused by the differences in sex composition. Finally, there are many measures to test the personality traits. However, the research on which one relates itself best to clinical outcomes after TKA is unknown at present. In a future study, we will take further steps to consider the aspects mentioned above.

## Conclusion

Our results may help clinicians identify patients at risk for high costs on the TKA for the public health system. Screening out these patients before surgery would help surgeons take preventive measures to significantly reduce the economic burden, avoid unnecessary suffering, and shorten the recovery period.
